# Public health round-up

**DOI:** 10.2471/BLT.22.010122

**Published:** 2022-01-01

**Malaria cases and deaths rise**

Two boys sleep under a mosquito net in Banalia, a small town in Tshopo province, Democratic Republic of the Congo, one of the countries to see estimated malaria cases rise in 2020. According to the World Health Organization’s *World malaria report*, published on 6 December, there were an estimated 241 million malaria cases in 2020, 14 million higher than were reported in 2019. Disruptions in the provision of malaria prevention, diagnosis and treatment during the pandemic are likely to have contributed to the negative trends.

**Figure:**
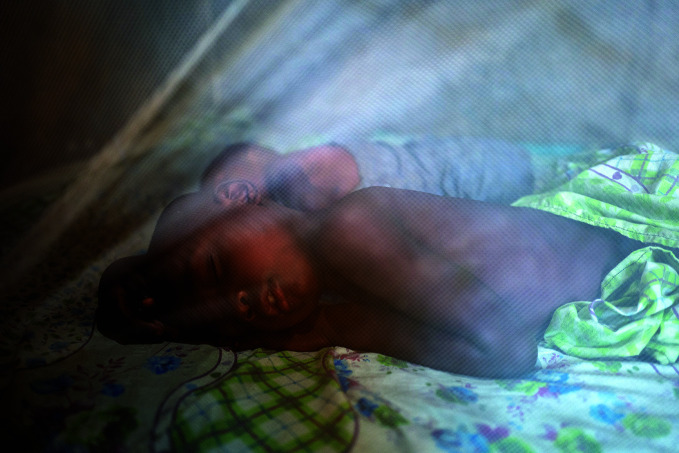
UNICEF/Gwenn Dubourthoumieu

## Omicron variant of concern

The first laboratory-confirmed case of a new severe acute respiratory syndrome coronavirus 2 (SARS-CoV-2) variant (B.1.1.529) was identified from a specimen collected on 9 November 2021 in South Africa. In consideration of the number of mutations (26–32) in the variant’s spike protein which may have implications for transmissibility and pathogenicity, as well as initial epidemiological reports, and at the advice of the Technical Advisory Group on Virus Evolution, the World Health Organization (WHO) classified it as a variant of concern (VOC) on 26 November.

On 2 December, WHO deployed a surge team to Gauteng Province, South Africa, where the first cluster was reported, to support surveillance, contact tracing, infection prevention and control measures and mobilized US$ 12 million to support critical response activities in countries across the region for a period of three months. As of 7 December, omicron cases had been reported in 57 countries across all WHO regions, including 337 confirmed cases in the European Union and European Economic Area.

As with other VOCs, accurate estimates of omicron distribution are complicated by variable surveillance capacity, including differences in sequencing capacities and sampling strategies between countries, as well as delays in reporting. At an 8 December briefing, WHO confirmed that existing diagnostic tests work for the new variant and called on Member States to increase surveillance, testing and gene sequencing. Multiple epidemiological and virological studies are underway and WHO is committed to communicating new findings with Member States and to the public as they are published.

https://bit.ly/3dySbxp

https://bit.ly/3dxeKCH

## Pandemic impacts malaria response

The coronavirus disease 2019 (COVID-19) pandemic disrupted malaria services, leading to a marked increase in cases and deaths in 2020. According to the WHO *World malaria report*, published on 6 December, there were an estimated 241 million malaria cases in 2020, 14 million more than were reported in 2019. Malaria deaths also rose, increasing by 69 000 to 627 000 malaria deaths worldwide in 2020. Approximately two thirds of these additional deaths (47 000) were linked to disruptions in the provision of malaria prevention, diagnosis and treatment during the pandemic.

Sub-Saharan Africa continues to carry the heaviest malaria burden, accounting for about 95% of all malaria cases and 96% of all deaths in 2020. About 80% of deaths in the region are among children under 5 years of age.

“Even before the COVID-19 pandemic struck, global gains against malaria had levelled off,” said WHO Director-General Tedros Adhanom Ghebreyesus. He pointed out that the numbers could have been worse had not countries stepped up response efforts. “Now, we need to harness that same energy and commitment to reverse the setbacks caused by the pandemic and step up the pace of progress against this disease,” he said.

https://bit.ly/3DCHhl6

## Gavi supports malaria vaccine rollout

Gavi, the Vaccine Alliance, is to support the introduction, procurement and delivery of the RTS,S malaria vaccine. On 2 December, the Gavi board announced an initial investment of US$ 155.7 million to cover the needs of Gavi-eligible countries in sub-Saharan Africa during the period 2022–2025.

https://bit.ly/3rMney7

## Towards a global accord on pandemics

The World Health Assembly agreed to initiate the global process required to draft and negotiate a convention, agreement or other international instrument under the WHO Constitution to strengthen pandemic prevention, preparedness and response.

Meeting in a Special Session, the second-ever since WHO’s founding in 1948, the Health Assembly adopted a sole decision titled, “The World Together,” on 1 December. The decision establishes an intergovernmental negotiating body (INB) that will draft and negotiate the international instrument with a view to its being adopted under Article 19 of the WHO Constitution, or other provisions of the Constitution as may be deemed appropriate by the INB.

The INB will hold its first meeting by 1 March 2022 to agree on ways of working and timelines and its second by 1 August 2022 to discuss progress on a working draft. It will also hold public hearings to inform its deliberations, deliver a progress report to the Seventy-sixth World Health Assembly in 2023, and submit its outcome for consideration by the Seventy-seventh World Health Assembly in 2024.

WHO Director-General Tedros Adhanom Ghebreyesus called the agreement a once-in-a-generation opportunity to strengthen the global health architecture to protect and promote the well-being of all people.

https://bit.ly/3Egmwwl

## COVID-19 vaccine donation concerns

The majority of donations of COVID-19 vaccines made to COVAX, the African Vaccine Acquisition Trust (AVAT), and African countries have been ad hoc and provided with little notice while the vaccines themselves have had short shelf lives, making it extremely challenging for countries to plan and implement vaccination campaigns.

This is according to a 29 November joint statement made by AVAT, the Africa Centres for Disease Control and Prevention (Africa CDC) and COVAX with a view to alerting the international community to the situation of donations of COVID-19 vaccines to Africa, and other COVAX participating economies, particularly those supported by the Gavi COVAX Advance Market Commitment (AMC).

The group called donors and manufacturers to ensure that donations are made in a way that allows countries to effectively mobilize domestic resources in support of rollout and enables long-term planning to increase coverage rates.

https://bit.ly/3IgJ3LW

## Licensing agreement for COVID-19 serological test

WHO’s COVID-19 Technology Access Pool (C-TAP) and the Medicines Patent Pool (MPP) finalized a licensing agreement with the Spanish National Research Council (CSIC) for a COVID-19 serological antibody test. The test effectively checks for the presence of SARS-CoV-2 antibodies developed either in response to a COVID-19 infection or to a vaccine.

Announced on 23 November, the agreement represents the first transparent, global, non-exclusive license for a COVID-19 health tool, and the first test license signed by MPP and included in the C-TAP.

The agreement covers all related patents and the biological material necessary for manufacture of the test and is aimed at facilitating the rapid manufacture and commercialization of the test worldwide. CSIC will provide all know-how to MPP and/or to prospective licensees as well as training. The license will be royalty-free for low- and middle-income countries and will remain valid until the date the last patent expires.

https://bit.ly/3r8g5b3

## Convalescent plasma treatment of COVID-19

WHO recommended that convalescent plasma not be used in the treatment of non-severe COVID-19 patients. The recommendation was part of a living guideline update on COVID-19 therapeutics released on 7 December.

An independent panel of experts considered pooled data from 16 randomized controlled trials, including 16 236 patients with non-severe, severe, and critical covid-19 infection and found that convalescent plasma does not improve survival or reduce the need for mechanical ventilation. Convalescent plasma is a transfusion of blood plasma from someone who has recovered from COVID-19.

https://bit.ly/3GmvPMc

**Cover photo**

Mother and child in El Muelle neighbourhood Bilwi, Puerto Cabezas, Nicaragua. The area was devastated by hurricane Eta which made landfall in Nicaragua on 3 November 2020.

**Figure:**
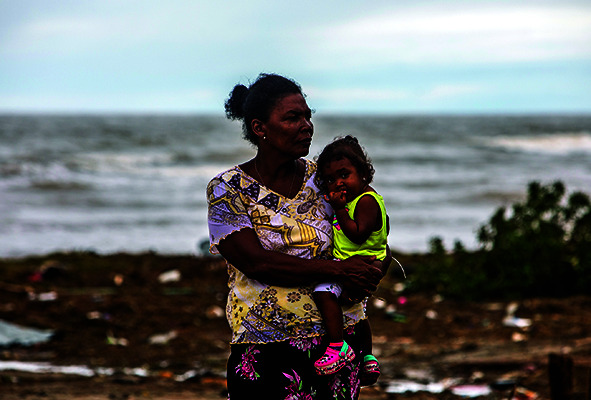
UNICEF/Gómez

## Schistosomiasis prevalence falls

The prevalence of schistosomiasis among school-aged children in sub-Saharan Africa fell by almost 60% in the past 20 years, the decline associated with the scaling up of preventive chemotherapy with praziquantel.

This is according to a spatiotemporal modelling study led by the Swiss Tropical and Public Health Institute in collaboration with WHO, the University of Basel and partners that was published on 3 December.

“The study highlights the effectiveness of the WHO-recommended strategy in treating school-aged children periodically,” said Dr Amadou Garba Djirmay of WHO’s Department of Control of Neglected Tropical Diseases. Garba Djirmay stressed, however, the need to implement integrated strategies covering water, sanitation and hygiene, snail control, education and awareness-raising.

https://bit.ly/3DkDF70

**Looking ahead**

30 January. World Neglected Tropical Diseases Day. https://bit.ly/3rq7OQ1

16–17 February. Global Disability Summit. https://bit.ly/3plrwd1

17–18 March. High-level meeting on health and migration in the WHO European Region.

https://bit.ly/3Des3T6

